# Proteomic Differences in Blood Plasma Associated with Antidepressant Treatment Response

**DOI:** 10.3389/fnmol.2017.00272

**Published:** 2017-08-31

**Authors:** Christoph W. Turck, Paul C. Guest, Giuseppina Maccarrone, Marcus Ising, Stefan Kloiber, Susanne Lucae, Florian Holsboer, Daniel Martins-de-Souza

**Affiliations:** ^1^Max Planck Institute of Psychiatry Munich, Germany; ^2^Laboratory of Neuroproteomics, Department of Biochemistry and Tissue Biology, Institute of Biology, University of Campinas Campinas, Brazil; ^3^Centre for Addiction and Mental Health Toronto, ON, Canada; ^4^Department of Psychiatry, University of Toronto Toronto, ON, Canada; ^5^HMNC GmbH Munich, Germany; ^6^Neurobiology Center, University of Campinas Campinas, Brazil

**Keywords:** major depressive disorder, antidepressant, symptom, response, plasma, biomarker, mass spectrometry

## Abstract

The current inability of clinical psychiatry to objectively select the most appropriate treatment is a major factor contributing to the severity and clinical burden of major depressive disorder (MDD). Here, we have attempted to identify plasma protein signatures in 39 MDD patients to predict response over a 6-week treatment period with antidepressants. LC-MS/MS analysis showed that differences in the levels of 29 proteins at baseline were found in the group with a favorable treatment outcome. Most of these proteins were components of metabolism or immune response pathways as well as multiple components of the coagulation cascade. After 6 weeks of treatment, 43 proteins were altered in responders of which 2 (alpha-actinin and nardilysin) had been identified at baseline. In addition, 46 proteins were altered in non-responders and 9 of these (alpha-actinin, alpha-2-macroglobulin, apolipoprotein B-100, attractin, C-reactive protein, fibrinogen alpha chain, fibrinogen beta chain, nardilysin and serine/threonine-protein kinase Chk1) had been identified at baseline. However, it should be stressed that the small sample size precludes generalization of the main results. Further studies to validate these as potential biomarkers of antidepressant treatment response are warranted considering the potential importance to the field of psychiatric disorders. This study provides the groundwork for development of novel objective clinical tests that can help psychiatrists in the clinical management of MDD through improved prediction and monitoring of patient responses to antidepressant treatments.

## Introduction

Major depressive disorder (MDD) is a leading cause of global disability and may affect about 20% during lifetime^1^^1^. MDD is a multivariate disorder presenting with a wide range of symptoms and the degree of treatment success varies among patients. About 40% of MDD patients do not respond to current treatments resulting in a high rate of individuals confronted with treatment resistance. In addition, MDD has a high relapse risk and results in premature death by suicide compared to the general population. Therefore, better treatment approaches are needed in this field of medicine, which has become an increasing global concern (Bromet et al., 2011).

The first-line therapy for patients with moderate to severe MDD is the administration of antidepressant medications (Chan et al., 2014). This treatment is often long and arduous as it can take several weeks before clinical response and efficacy of antidepressant treatment can be determined. Patients who show no response often have to endure one or more additional treatment trials, which may involve dose increase, augmentation with compounds of the same or other drug classes, or switching of antidepressants. Furthermore, approximately 25% of patients who do not improve with the first treatment discontinue their medication (Chan et al., 2014). Nonadherence is also a significant predictor of negative outcomes for patients with major affective disorders (Pompili et al., 2013). This can lead to high rates of recurrence, hospitalization, functional impairment, active suicidal ideation and suicidal behavior. A history of nonadherence to commonly available antidepressant medications has been associated with a more negative outcome in major depression, increased suicide attempts and enhanced active suicidal ideation. Therefore, improving adherence to antidepressant drugs may generally help clinicians to prevent both major depression and suicidal behaviors. In line with this objective, the identification of biological indicators that could be used in a test to predict treatment response would help to improve outcomes, decrease length of treatment and number of insufficient treatment trials with tremendous impact on lives of patients, healthcare and societal costs.

Recent studies have described the investigation of psychiatric disorders through proteomic analysis of peripheral body fluids, such as blood plasma or serum (Schwarz et al., 2012a; Stelzhammer et al., 2014; van Beveren et al., 2014; Chan et al., 2015; Jordan et al., 2016) and have used proteomic analyses of plasma or serum to identify molecular signatures that can be used to predict response to antipsychotic treatments in patients with schizophrenia (Schwarz et al., 2012b, 2015; Tomasik et al., 2016). Changes in peripheral body fluids can reflect processes in the brain due to the two-way communication between the brain and periphery and the blood serving as a conduit through the transport of bioactive molecules (Guest et al., 2015). Such bioactive molecules include proteins, such as hormones, immune and inflammatory factors, metabolism-related proteins, and transport proteins. As an example, alterations in peripheral cytokine levels can also affect the brain and influence the synthesis, release and reuptake of mood-relevant neurotransmitters (Chan et al., 2014). In addition, a number of reports have shown that impairments in hypothalamic-pituitary-adrenal (HPA) axis function and insulin signaling can contribute to inflammation, neurological dysfunctions and memory deficits and circulating levels of growth factors, such as insulin-like growth factor-1 (IGF-1) brain-derived neurotrophic factor (BDNF) and leptin can have influence on mood and cognitive brain function (Chan et al., 2014).

Here, we present a pilot study to determine the possibility to detect plasma proteins that can be used for response prediction in MDD patients prior to treatment with antidepressants. This is an open-label naturalistic clinical study, all patients are inpatients who received single or combined treatment with several types of medication. We have used liquid chromatography tandem mass spectrometry (LC-MS/MS) with label-free spectral counting to identify proteins in plasma from these individuals as described in two recent studies of schizophrenia (Martins-de-Souza et al., 2015; Saia-Cereda et al., 2015). In addition, we were interested to identify proteins showing changes in concentration depending on response to treatment. LC-MS/MS was chosen given the wider range of coverage compared to other platforms, in terms of total numbers of identifiable proteins. In addition, plasma was chosen over the use of serum due to the wider number of clotting factors present in the former (Alsaif et al., 2012).

A previous LC-MS/MS profiling study found that the levels of 5 serum proteins could be used to distinguish between MDD and control groups (Lee et al., 2016). In addition, a multiplex immunoassay profiling analysis of serum identified several proteins that may be involved in the pathophysiology of depression (Frye et al., 2015) and another such study identified a 9-plex panel that could be used to predict response to different antidepressants (Chan et al., 2016). The present LC-MS/MS profiling study could provide insights in the molecular pathways affected in both responders and non-responders and thereby lay the groundwork for development of tests for guiding treatment and for detection of novel therapeutics for MDD. It should be noted that the small sample size and preliminary nature of the current study obviates generalization of the findings to wider patient groups.

## Materials and methods

### Subjects

The study was approved by the Ethics Committee of the Medical Faculty of the Ludwig Maximilians University in Munich, Germany. Plasma was collected from 39 patients suffering from a current a major depressive episode, who participated in the Munich Antidepressant Response Signature (MARS) project (Hennings et al., 2009; Table 1). MARS is an open-label naturalistic clinical study and inpatients admitted to the clinic of the Max Planck Institute of Psychiatry for treatment for depression were included. Details of the study were explained and a written informed consent was obtained from each patient. Patients were diagnosed by trained psychiatrists according to DSM-IV criteria. Only patients with single or recurrent major depression with at least moderate depression severity were included. This required a baseline score of 14 or more on the 21 item version of the Hamilton Depression Rating Scale (HAM-D) (Hamilton, 1960). All patients selected for this analysis had a HAM-D score ≥20 at study entry. All patients of this study were not taking any anti-inflammatory or immunosuppresant medications when admitted to the clinic. Only three patients were using nonsteroidal anti-inflammatory drugs (Supplementary Table 1). No cases of bipolar disorder were included in this analysis. Patients received single or combined treatment with several types of medication including selective serotonin reuptake inhibitors (SSRIs), serotonin–norepinephrine reuptake inhibitor (SNRIs), tricyclic antidepressants (TCA), or dual noradrenergic and serotonergic antidepressants (NASSA, SNRI). Combination therapy of two antidepressants and augmentation with mood stabilizers or second generation antipsychotics was allowed. Treatment outcome was evaluated with weekly HAM-D assessments. Response was defined as a reduction of 50% or more of the baseline HAM-D score after 6 weeks of treatment (T6). Of the 39 patients, 25 were classified as responders and 14 as non-responders according to the criteria set out above. As expected there was a significant difference in T6 HAM-D scores between responders and non-responders (*P* < 0.001). No significant differences were observed between responders and non-responders with regards to gender (*P* = 1.000; two-sided Fisher's exact test), age (*P* = 0.563; Mann Whitney *U*-test), body mass index (*P* = 0.189; Mann-Whitney) or HAM-D scores at baseline (*P* = 0.664). None of the study subjects had diabetes.

**Table 1:** Demographics.

	**Responders**	**Non-responders**	***P*-value**
Gender (male/female)	13/11	8/7	1.000
Age (years)	45.0 ± 17.6	47.5 ± 17.1	0.563
Body mass index (kg/m^2^)	23.5 ± 3.2	24.8 ± 2.8	0.189
Age at disease onset	36.7 ± 17.9	33.2 ± 16.3	0.555
No. of previous episodes	1.5 ± 1.4	5.2 ± 6.9	0.082
Diagnosis (single episode/recurrent depression)	6/18	3/12	0.718
Ham-D T0	28.9 ± 6.3	27.9 ± 4.4	0.664
Ham-D T6	7.0 ± 5.3	21.7 + 5.6	*P* < 0.001

### Blood plasma samples

Fasting venous blood was collected in the morning in sample tubes containing potassium EDTA immediately after clinical admission and again after 6 weeks of pharmacological treatment. Plasma was separated from blood using the Accuspin System Hystopaque™-1077 (Sigma Aldrich, Taufkirchen, Germany) according to the manufacturer's protocol and protein concentrations were determined using the Bradford assay (Bio-Rad; Hercules, CA, USA).

### Proteomics

Plasma samples were depleted of high-abundance proteins using the MARS-14 immunodepletion system (Agilent; Wokingham, UK) since these can disrupt resolution of lower abundance proteins in mass spectrometry-based proteomic studies. The depleted flow-through fractions were first treated with 5 mM dithiothreitol (30 min, room temperature) to reduce protein disulfide bonds and then with 10 mM iodoacetamide (30 min, 60°C in the dark) to alkylate the reactive sulfhydryl groups. Each sample was proteolytically digested with trypsin (Promega; Heidelberg, Germany) overnight (1:80 trypsin: protein ratio) for 16 h at 37°C. The resulting peptides were lyophilized and frozen prior to mass spectrometry analyses. Immediately prior to analysis, peptides were dissolved in 0.1% formic acid and injected into the nano-liquid chromatography (LC) system consisting of an autosampler and two-dimensional (2D)-nano high-performance liquid chromatography (HPLC; Eksigent, Dublin, CA, USA), coupled online to an LTQ-Orbitrap XL mass spectrometer (Thermo Scientific; Bremen, Germany). The full detailed description of the nano LC–MS/MS configuration and data analyses can be found in Maccarrone et al. (2013).

For mass spectrometry analysis, samples were measured in data dependent acquisition mode. Survey scans were acquired at a resolution of 60,000 and the 10 most intense signals were fragmented using collision-induced dissociation. The target values and maximum injection times were 10^6^ ions and 100 ms for MS mode and 10^4^ ions and 50 ms for MS/MS mode, respectively. Precursor ions with charge states between 2^+^ and 5^+^ were selected for fragmentation, using a 15 s dynamic exclusion. Raw data were processed using an in-house version of the MASCOT search engine for protein identification and MASCOT Distiller (Matrix Sciences; London, UK) for label-free spectral counting quantification. The cut-off criteria were set at a minimum of 2 peptides for identification and at least 5 MS/MS spectra for quantification to increase the stringency of the process. Differences in protein expression between responders and non-responders (R/NR) at baseline (T0) and between T6 and T0 (T6/T0) were determined using Student's *t*-test (*P* < 0.05). A cut-off of 50% (>1.50, <0.66) difference was used as previously established for this workflow (Maccarrone et al., 2013). Proteins present at differential levels were classified according to their biological and molecular processes using the Human Protein Reference Database (http://www.hprd.org). Note that a maximum ratio of >5.00 and <0.20 was used due to limitations of the algorithm employed.

It should also be noted that our objective was to observe common protein signatures of antidepressant response and biological modulation and therefore the type of treatment, e.g., antidepressant class, augmentation, was not taken into consideration.

## Results and discussion

### Identification of proteins at baseline (T0) by comparing responders and non-responders

Plasma samples taken from patients at baseline (T0) were separated into responders (*n* = 25) and non-responders (*n* = 14), as determined by whether or not they showed a 50% reduction in symptoms at the end of the 6 week treatment period. The proteomic comparison of samples from responder and non-responders led to identification of 29 proteins that showed significant differences (*P* < 0.05; > ±50%) in their plasma levels and could therefore be predictive of response (Table 2). The most prevalent biological processes associated with these proteins were protein metabolism (*n* = 9) and immune response (*n* = 9), followed by energy metabolism (*n* = 3), transport (*n* = 3), cell growth and maintenance (*n* = 3) and cell communication and signaling (*n* = 2). Previous proteomic investigations have implicated these pathways in acute MDD (Chan et al., 2014; Stelzhammer et al., 2014; Young et al., 2014; Liu et al., 2015).

**Table 2:** Proteins present at different concentrations in plasma at baseline (T0) associated with a favorable response to subsequent treatment for 6 weeks with antidepressants.

**UniPort**	**Protein name**	**Gene**	**R/NR**	**Biological process**	**Molecular class**	**Molecular function**
P12814	Alpha-actinin-1	ACTN1	>5.00	Cell growth and maintenance	Cytoskeletal associated protein	Cytoskeletal protein binding
P02741	C-reactive protein	CRP	>5.00	Immune response	Complement protein	Complement activity
P07996	Thrombospondin-1	THBS1	3.52	Cell growth and maintenance	Extracellular matrix protein	Extracellular matrix structural constituent
P02671	Fibrinogen alpha chain	FGA	2.98	Protein metabolism	Coagulation factor	Protein binding
Q9BXR6	Complement factor H-related protein 5	CFHR5	2.57	Immune response	Secreted polypeptide	Complement binding
P53367	Arfaptin-1	ARFIP1	2.50	Cell communication and Signaling	Unclassified	Regulator of G-protein signaling activity
P12259	Coagulation factor V	F5	2.50	Protein metabolism	Coagulation factor	Metal ion binding
P36980	Complement factor H-related protein 2	CFHR2	2.20	Immune response	Secreted polypeptide	Molecular function unknown
Q9NUI1	Peroxisomal 2,4-dienoyl-CoA reductase	DECR2	1.83	Metabolism	Enzyme: Reductase	Catalytic activity
P32119	Peroxiredoxin-2	PRDX2	1.75	Metabolism	Enzyme: Peroxidase	Peroxidase activity
Q03591	Complement factor H-related protein 1	CFHR1	1.65	Immune response	Complement protein	Complement activity
P02743	Serum amyloid P-component	APCS	1.65	Protein metabolism	Secreted polypeptide	Binding
P60709	Actin, cytoplasmic 1	ACTB	1.63	Cell growth and maintenance	Cytoskeletal protein	Structural constituent of cytoskeleton
P02675	Fibrinogen beta chain	FGB	1.55	Protein metabolism	Coagulation factor	Protein binding
O43866	CD5 antigen-like	CD5L	1.52	Immune response	Secreted polypeptide	Defense/immunity protein activity
P00742	Coagulation factor X	F10	0.65	Protein metabolism	Coagulation factor	Peptidase activity
P05155	Plasma protease C1 inhibitor	SERPING1	0.64	Protein metabolism	Protease inhibitor	Protease inhibitor activity
O75882	Attractin	ATRN	0.62	Immune response	Defensin	Defense/immunity protein activity
P22352	Glutathione peroxidase 3	GPX3	0.61	Metabolism	Enzyme: Peroxidase	Peroxidase activity
P02760	Protein AMBP	AMBP	0.60	Immune response	Secreted polypeptide	Defense/immunity protein activity
P04114	Apolipoprotein B-100	APOB	0.59	Transport	Transport/cargo protein	Transporter activity
P02753	Retinol-binding protein 4	RBP4	0.59	Transport	Transport/cargo protein	Transporter activity
P25311	Zinc-alpha-2-glycoprotein	AZGP1	0.58	Immune response	Adhesion molecule	Cell adhesion molecule activity
P06727	Apolipoprotein A-IV	APOA4	0.47	Transport	Transport/cargo protein	Transporter activity
Q96IY4	Carboxypeptidase B2	CPB2	0.45	Protein metabolism	Carboxypeptidase	Carboxypeptidase activity
P01023	Alpha-2-macroglobulin	A2M	0.44	Protein metabolism	Protease inhibitor	Protease inhibitor activity
P10643	Complement component C7	C7	0.40	Immune response	Complement protein	Complement activity
O14757	Serine/threonine-protein kinase Chk1	CHEK1	<0.20	Cell communication and signaling	Serine/threonine kinase	Protein serine/threonine kinase activity
O43847	Nardilysin	NRD1	<0.20	Protein metabolism	Metallo protease	Metallopeptidase activity

*R/NR, response/non-response ratio. Values were capped at ratios of >5.0 and <0.2 due to the ion counting algorithm used. Increased values are shaded in light gray and decreased values are in dark gray*.

The greatest differences were found for alpha-actinin-1, C-reactive protein, thrombospondin-1, fibrinogen alpha chain, complement factor H-related protein 5, coagulation factor V, arfaptin-1 and complement factor H-related protein 2, which were all increased by more than 2-fold in responders. Most of these proteins are related to the innate immune response, inflammation and the coagulation cascade. Effects on protein components of these pathways have been reported previously for MDD patients at risk of suicide (Yang et al., 2016) and as markers of treatment resistance (Ruland et al., 2016). Furthermore, in a previous study, we found that levels of fibrinogen, a key component of the clotting cascade, may be predictive of antidepressant treatment response (Martins-de-Souza et al., 2014). As further evidence for effects on the coagulation cascade, a recent study showed that major depression patients at baseline had an enhanced aggregating response to arachidonic acid and increased levels of clotting factors, such as fibrinogen and factor V (Holzer et al., 2012).

Four proteins were by more than 2-fold lower in responders, i.e., apolipoprotein A-IV, carboxypeptidase B2, complement component C7 and serine/threonine-protein kinase Chk1. In addition, three proteins associated with a transport function were lower including apolipoprotein A-IV, apolipoprotein B-100 and retinol-binding protein 4. Previous studies have identified lower levels of all of these proteins in major depression patients (Lopez-Vilchez et al., 2014; Frye et al., 2015; Lee et al., 2016; Ruland et al., 2016; Kim et al., 2017). In addition, the finding of lower levels of apolipoprotein A-IV in responders is consistent with the findings of a recent study showing that this protein may be a marker of response to different antidepressants (Ruland et al., 2016). The changes in the other proteins may represent novel findings requiring further validation.

### Identification of protein changes after 6 weeks in responders

Plasma samples taken after 6 weeks of treatment with antidepressants were compared with those taken at baseline (T0) to identify protein changes associated with response to treatment. This led to identification of 43 proteins (Table 3). Of those proteins, 18 increased more than 2-fold compared to baseline. One of these proteins was putative peptide YY-2 which is consistent with previous studies reporting that peptide YY levels are altered in patients with MDD (Giménez-Palop et al., 2012) and bipolar disorder (Haenisch et al., 2015). In addition, peptide YY is thought to be involved in regulation of food intake, circadian rhythms, cognition and behavior (Śliwińska-Mossoń et al., 2013). Another protein which showed a large increase (>5-fold) after treatment in responders was serine/threonine-protein kinase Chk1, which may reflect a treatment-normalizing effect since levels were low (R/NR < 0.20) at baseline. Further studies including follow up validation will be required to corroborate these findings. In addition, two other kinases (serine/threonine-protein kinase Nek1 and microtubule-associated serine/threonine-protein kinase 4) also showed an increase >2-fold after antidepressant treatment in responders. This could suggest a general effect on kinases involved in a positive response to treatment.

**Table 3:** Proteins present at different concentrations in plasma associated with a favorable response to treatment for 6 weeks with antidepressants.

**UniPort**	**Protein name**	**Gene**	**T6/T0**	**Biological process**	**Molecular class**	**Molecular function**
Q9NRI6	Putative peptide YY-2	CD5L PYY2	>5.00	Unknown	Unclassified	Molecular function unknown
P50135	Histamine N-methyltransferase	HNMT	>5.00	Metabolism	Enzyme: Methyltransferase	Methyltransferase activity
Q9BT25	HAUS augmin-like complex subunit 8	HAUS8	>5.00	Unknown	Unclassified	Molecular function unknown
Q9Y2J0	Rabphilin-3A	RPH3A	>5.00	Transport	Membrane transport protein	Auxiliary transport protein activity
P37198	Nuclear pore glycoprotein p62	NUP62	>5.00	Transport	Transport/cargo protein	Transporter activity
O14757	Serine/threonine-protein kinase Chk1	CHEK1	>5.00	Cell communication	Serine/threonine kinase	Protein serine/threonine kinase activity
Q8IV08	Phospholipase D3	PLD3	>5.00	Metabolism	Enzyme: Phospholipase	Phospholipase activity
Q9NPP4	NLR family CARD domain-containing protein 4	NLRC4	>5.00	Cell communication	Adapter molecule	Receptor signaling complex scaffold activity
O43847	**Nardilysin**	NRD1	>5.00	Protein metabolism	Metallo protease	Metallopeptidase activity
O14727	Apoptotic protease-activating factor 1	APAF1	>5.00	Cell growth and maintenance	Adapter molecule	Receptor signaling complex scaffold activity
Q96GQ7	Probable ATP-dependent RNA helicase DDX27	DDX27	>5.00	Reg. nucleic acid metabolism	RNA helicase	Helicase activity
Q96FV9	THO complex subunit 1	THOC1	>5.00	Reg. nucleic acid metabolism	Transcription regulatory protein	Transcription regulator activity
Q96PY6	Serine/threonine-protein kinase Nek1	NEK1	>5.00	Cell communication	Serine/threonine kinase	Protein serine/threonine kinase activity
Q96KD3	Protein FAM71F1	FAM71F1	>5.00	Unknown	Unclassified	Molecular function unknown
Q92750	Transcription initiation factor TFIID subunit 4B	TAF4B	>5.00	Cell growth and maintenance	Transcription factor	Transcription factor activity
P12814	**Alpha-actinin-1**	ACTN1	3.02	Cell growth and maintenance	Cytoskeletal associated protein	Cytoskeletal protein binding
O15021	Microtubule-associated serine/threonine-protein kinase 4	MAST4	2.65	Unknown	Unclassified	Molecular function unknown
P04075	Fructose-bisphosphate aldolase A	ALDOA	2.00	Metabolism	Enzyme: Lyase	Lyase activity
P02751	Fibronectin	FN1	1.68	Cell growth and maintenance	Extracellular matrix protein	Extracellular matrix structural constituent
P23142	Fibulin-1	FBLN1	1.58	Cell growth and maintenance	Extracellular matrix protein	Extracellular matrix structural constituent
P08603	Complement factor H	CFH	0.67	Immune response	Regulatory/other subunit	Complement binding
P04217	Alpha-1B-glycoprotein	A1BG	0.58	Unknown	Secreted polypeptide	Molecular function unknown
Q9NUI1	Peroxisomal 2,4-dienoyl-CoA reductase	DECR2	0.57	Metabolism	Enzyme: Reductase	Catalytic activity
Q9UK55	Protein Z-dependent protease inhibitor	SERPINA10	0.43	Protein metabolism	Protease inhibitor	Protease inhibitor activity
Q5SNV9	Uncharacterized protein C1orf167	C1orf167	0.40	Unknown	Unclassified	Molecular function unknown
P46821	Microtubule-associated protein 1B	MAP1B	0.39	Cell growth and maintenance	Cytoskeletal associated protein	Cytoskeletal protein binding
P61769	Beta-2-microglobulin	B2M	0.39	Immune response	MHC complex protein	MHC class I receptor activity
Q9UKX2	Myosin-2	MYH2	0.26	Cell growth and maintenance	Structural protein	Structural molecule activity
Q9NUV9	GTPase IMAP family member 4	GIMAP4	0.25	Cell communication	GTPase	GTPase activity
Q99996	A-kinase anchor protein 9	AKAP9	<0.20	Cell communication	Adapter molecule	Receptor signaling complex scaffold activity
Q9BXL6	Caspase recruitment domain-containing protein 14	CARD14	<0.20	Cell growth and maintenance	Enzyme: Phosphotransferase	Catalytic activity
Q96PC5	Melanoma inhibitory activity protein 2	MIA2	<0.20	Unknown	Unclassified	Molecular function unknown
Q96MW7	Tigger transposable element-derived protein 1	TIGD1	<0.20	Reg. nucleic acid metabolism	DNA binding protein	DNA binding
P46100	Transcriptional regulator ATRX	ATRX	<0.20	Reg. nucleic acid metabolism	Transcription regulatory protein	Transcription regulator activity
Q96DA0	Zymogen granule protein 16 homolog B	ZG16B	<0.20	Unknown	Unclassified	Molecular function unknown
Q13813	Spectrin alpha chain, non-erythrocytic 1	SPTAN1	<0.20	Cell growth and maintenance	Cytoskeletal protein	Structural constituent of cytoskeleton
Q9UPN3	Microtubule-actin cross-linking factor 1, isoforms 1/2/3/5	MACF1	<0.20	Cell growth and maintenance	Structural protein	Structural molecule activity
Q96RL7	Vacuolar protein sorting-associated protein 13A	VPS13A	<0.20	Protein metabolism	Transport/cargo protein	Transporter activity
Q9Y2J2	Band 4.1-like protein 3	EPB41L3	<0.20	Cell growth and maintenance	Structural protein	Structural molecule activity
Q9NSC5	Homer protein homolog 3	HOMER3	<0.20	Cell communication	Adapter molecule	Receptor signaling complex scaffold activity
P55060	Exportin-2	CSE1L	<0.20	Transport	Transport/cargo protein	Transporter activity
Q09666	Neuroblast differentiation-associated protein AHNAK	AHNAK	<0.20	Metabolism	Unclassified	Protein binding
Q70E73	Ras-associated and pleckstrin homology domains-containing protein 1	RAPH1	<0.20	Cell growth and maintenance	Cytoskeletal associated protein	Cytoskeletal protein binding

*T6/T0, the ratio of the protein levels at 6 weeks to the baseline values. Values were capped at ratios of >5.0 and <0.2 due to the ion counting algorithm used. Increased values are shaded in light gray and decreased values are in dark gray. Proteins indicated in bold were also identified at baseline*.

Of the remaining proteins, 14 showed a decrease >2-fold after antidepressant treatment. A large decrease (T6/T0 ratio = 0.26) in plasma mysosin-2 levels was observed in responders after treatment. As mysosin is a component of the cytoskeleton, the decreased levels of this protein may represent a lower level of tissue cellular damage.

Two of the proteins (alpha actinin 1 and nardilysin) altered by the treatment were also present at different levels in the responders at baseline (Table 4). Alpha actinin-1 showed similar changes in the same direction at T6 as seen at T0 although nardilysin showed opposite changes. Nardilysin is a metalloprotease that cleaves proproteins, such as dynorphin-A, α-neoendorphin, and glucagon at the N-terminus of arginine and lysine residues at sites on the proteins marked by dibasic amino acids. As such nardilysin is involved in proteolytic activation of hormones and neuropeptides involved in brain function. Previous studies have suggested that this endoprotease may be altered in Alzheimer disease, Down syndrome, schizophrenia, mood disorders, alcohol abuse, heroin addiction, and cancer (Bernstein et al., 2013).

**Table 4:** Proteins altered in responders (T6-R) and non-responders (T6-NR) after 6 weeks treatment with antidepressants compared with the values at baseline (T0-R/NR).

**UniProt**	**Protein name**	**Gene**	**T0–R/NR**	**T6-R**	**T6-NR**	**Biological process**
P12814	Alpha-actinin-1	ACTN1	>5.00	3.02	2.32	Cell growth and maintenance
P02741	C-reactive protein	CRP	>5.00		1.66	Immune response
P02671	Fibrinogen alpha chain	FGA	2.98		1.84	Protein metabolism
P02675	Fibrinogen beta chain	FGB	1.55		1.94	Protein metabolism
O75882	Attracting	ATRN	0.62		2.08	Immune response
P04114	Apolipoprotein B-100	APOB	0.59		0.33	Transport
P01023	Alpha-2-macroglobulin	A2M	0.44		0.64	Protein metabolism
O43847	Nardilysin	NRD1	<0.20	>5.00	<0.20	Protein metabolism
O14757	Serine/threonine-protein kinase Chk1	CHEK1	<0.20		3.42	Cell communication and signaling

*Values were capped at ratios of >5.0 and <0.2 due to the ion counting algorithm used. Increased values are shaded in light gray and decreased values are in dark gray*.

### Identification of proteins changes after 6 weeks in non-responders

After treatment 46 proteins were present at different levels compared to the T0 values in samples from patients who showed a poor clinical response (Table 5). Of these proteins, 25 were increased. At this stage, it is not possible to determine which of these changes are due to either drug effects, side effects or the poor response and further studies are warranted to determine this. Nine of these proteins were also present at different levels at baseline and the treatment appears to have restored two of these (attractin and serine/threonine-protein kinase Chk1) to normal levels (Table 4). It should be noted that the increase in serine/threonine-protein kinase Chk1 was smaller than that seen in the responders.

**Table 5:** Proteins present at different concentrations in plasma associated with non-response to treatment with antidepressants after 6 weeks.

**UniPort**	**Protein name**	**Gene**	**T6/T0**	**Biological process**	**Molecular class**	**Molecular function**
Q8IV08	Phospholipase D3	PLD3	>5.00	Metabolism	Enzyme: Phospholipase	Phospholipase activity
Q9Y5H0	Protocadherin gamma-A3	PCDHGA3	>5.00	Cell growth and maintenance	Adhesion molecule	Cell adhesion molecule activity
Q9NWV8	BRISC and BRCA1-A complex member 1	BABAM1	>5.00	Unknown	Unclassified	Molecular function unknown
O94913	Pre-mRNA cleavage complex 2 protein Pcf11	PCF11	>5.00	Reg. nucleic acid metabolism	RNA binding protein	RNA binding
P48552	Nuclear receptor-interacting protein 1	NRIP1	>5.00	Reg. nucleic acid metabolism	Transcription regulatory protein	Transcription regulator activity
Q9UK55	Protein Z-dependent protease inhibitor	SERPINA10	>5.00	Protein metabolism	Protease inhibitor	Protease inhibitor activity
P02751	Fibronectin	FN1	4.97	Cell growth and maintenance	Extracellular matrix protein	Extracellular matrix structural constituent
Q9UQP3	Tenascin-N	TNN	3.66	Cell growth and maintenance	Extracellular matrix protein	Extracellular matrix structural constituent
Q86W92	Liprin-beta-1	PPFIBP1	3.56	Cell communication	Anchor protein	Cytoskeletal anchoring activity
O14757	**Serine/threonine-protein kinase Chk1**	CHEK1	3.42	Cell communication	Serine/threonine kinase	Protein serine/threonine kinase activity
Q9BT25	HAUS augmin-like complex subunit 8	HAUS8	2.62	Unknown	Unclassified	Molecular function unknown
Q9NPP4	NLR family CARD domain-containing protein 4	NLRC4	2.45	Cell communication	Adapter molecule	Receptor signaling complex scaffold activity
Q1MSJ5	Centrosome and spindle pole-associated protein 1	CSPP1	2.41	Cell growth and maintenance	Unclassified	Molecular function unknown
P12814	**Alpha-actinin-1**	ACTN1	2.32	Cell growth and maintenance	Cytoskeletal associated protein	Cytoskeletal protein binding
Q9Y490	Talin-1	TLN1	2.22	Cell growth and maintenance	Cytoskeletal associated protein	Cytoskeletal protein binding
Q92954	Proteoglycan 4	PRG4	2.20	Cell growth and maintenance	Cell junction protein	Cell adhesion molecule activity
P04278	Sex hormone-binding globulin	SHBG	2.11	Transport	Transport/cargo protein	Transporter activity
O75882	**Attractin**	ATRN	2.08	Immune response	Defensin	Defense/immunity protein activity
P02675	**Fibrinogen beta chain**	FGB	1.94	Protein metabolism	Coagulation factor	Protein binding
P02671	**Fibrinogen alpha chain**	FGA	1.87	Protein metabolism	Coagulation factor	Protein binding
P26927	Hepatocyte growth factor-like protein	MST1	1.77	Cell communication	Growth factor	Growth factor activity
P02679	Fibrinogen gamma chain	FGG	1.76	Protein metabolism	Coagulation factor	Protein binding
P02741	**C-reactive protein**	CRP	1.66	Immune response	Complement protein	Complement activity
Q04756	Hepatocyte growth factor activator	HGFAC	1.61	Protein metabolism	Serine protease	Serine-type peptidase activity
Q06033	Inter-alpha-trypsin inhibitor heavy chain H3	ITIH3	1.59	Protein metabolism	Protease inhibitor	Protease inhibitor activity
P01023	**Alpha-2-macroglobulin**	A2M	0.64	Protein metabolism	Protease inhibitor	Protease inhibitor activity
P61769	Beta-2-microglobulin	B2M	0.48	Immune response	MHC complex protein	MHC class I receptor activity
Q9Y2J2	Band 4.1-like protein 3	EPB41L3	0.44	Cell growth and maintenance	Structural protein	Structural molecule activity
P04114	**Apolipoprotein B-100**	APOB	0.33	Transport	Transport/cargo protein	Transporter activity
P37198	Nuclear pore glycoprotein p62	NUP62	0.32	Transport	Transport/cargo protein	Transporter activity
Q9Y2J0	Rabphilin-3A	RPH3A	0.28	Transport	Membrane transport protein	Auxiliary transport protein activity
Q96MW7	Tigger transposable element-derived protein 1	TIGD1	0.24	Reg. nucleic acid metabolism	DNA binding protein	DNA binding
P55060	Exportin-2	CSE1L	0.23	Transport	Transport/cargo protein	Transporter activity
O60934	Nibrin	NBN	<0.20	Cell growth and maintenance	DNA repair protein	DNA binding
Q96PC5	Melanoma inhibitory activity protein 2	MIA2	<0.20	Unknown	Unclassified	Molecular function unknown
Q9H3Q3	Galactose-3-O-sulfotransferase 2	GAL3ST2	<0.20	Metabolism	Enzyme: Sulphotransferase	Sulfotransferase activity
O14727	Apoptotic protease-activating factor 1	APAF1	<0.20	Cell growth and maintenance	Adapter molecule	Receptor signaling complex scaffold activity
Q9UBP8	Kidney-associated antigen 1	KAAG1	<0.20	Immune response	Unclassified	Molecular function unknown
Q8WU90	Zinc finger CCCH domain-containing protein 15	ZC3H15	<0.20	Unknown	DNA binding protein	DNA binding
Q09666	Neuroblast differentiation-associated protein AHNAK	AHNAK	<0.20	Metabolism	Unclassified	Protein binding
O43847	**Nardilysin**	NRD1	<0.20	Protein metabolism	Metallo protease	Metallopeptidase activity
Q9UPN3	Microtubule-actin cross-linking factor 1, isoforms 1/2/3/5	MACF1	<0.20	Cell growth and maintenance	Cell growth and maintenance	Structural protein
Q13813	Spectrin alpha chain, non-erythrocytic 1	SPTAN1	<0.20	Cell growth and maintenance	Cytoskeletal protein	Structural constituent of cytoskeleton
O75716	Serine/threonine-protein kinase 16	STK16	<0.20	Cell communication	Serine/threonine kinase	Protein serine/threonine kinase activity
Q9BXL6	Caspase recruitment domain-containing protein 14	CARD14	<0.20	Cell growth and maintenance	Enzyme: Phosphotransferase	Catalytic activity
O15021	Microtubule-associated serine/threonine-protein kinase 4	MAST4	<0.20	Unknown	Unclassified	Molecular function unknown

*T6/T0, the ratio of the protein levels at 6 weeks to the baseline values. Values were capped at ratios of >5.0 and <0.2 due to the ion counting algorithm used. Increased values are shaded in light gray and decreased values are in dark gray. Proteins indicated in bold were also identified at baseline*.

**Figure 1:**
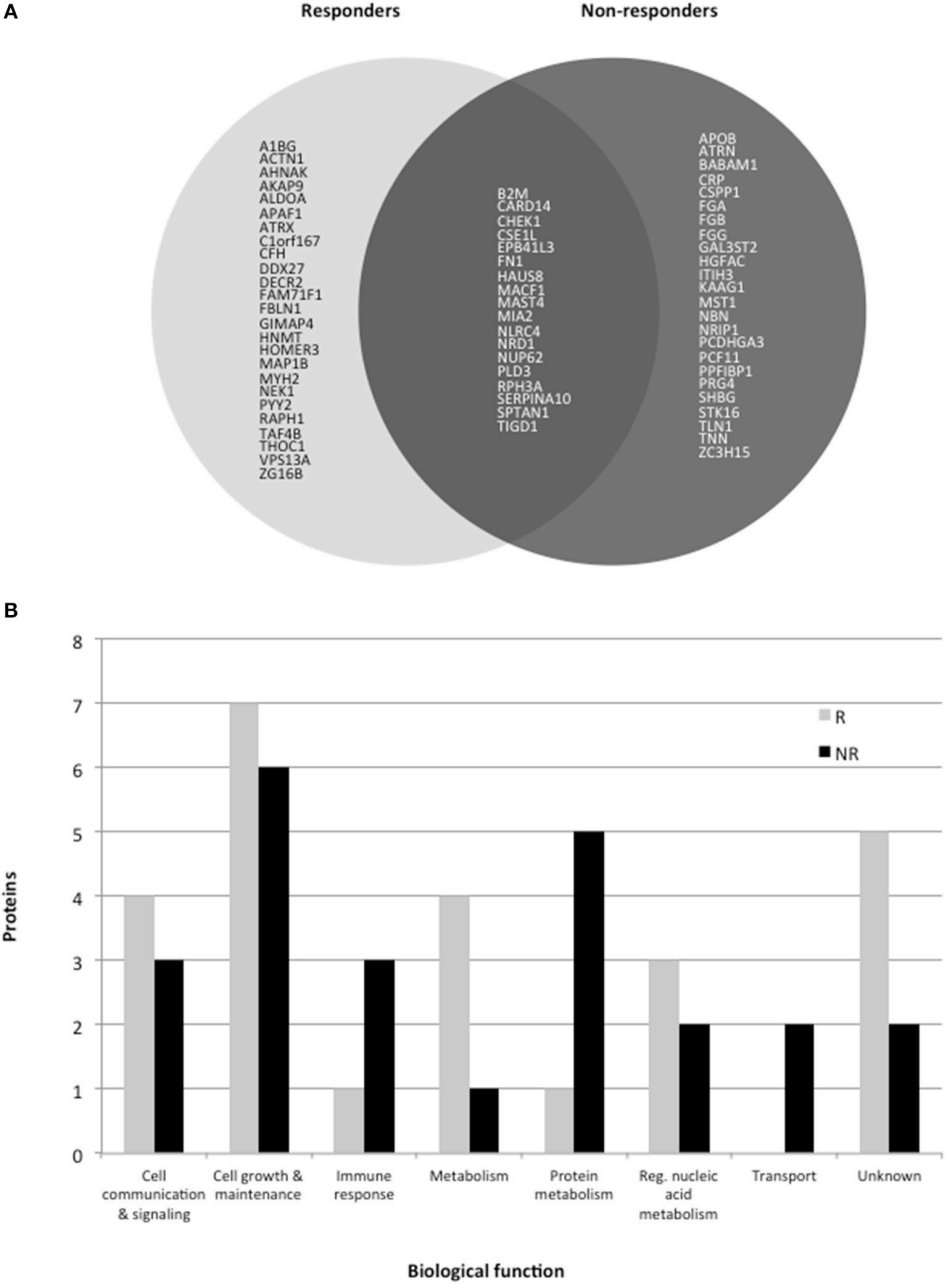
**(A)** Pie chart showing protein levels associated with response or non-response to treatment with antidepressants for 6 weeks. Only gene codes are shown (see tables for the full protein names. **(B)** Histogram showing the major biological processes altered in responders (R) and non-responders (NR).

Comparison of the main biological processes associated with each protein altered in responders and non-responders showed these were mostly similar (Figure 1). Eighteen proteins were altered in common which could be due to a common mechanism of action across the different antidepressants. However, more proteins associated with the immune response and protein metabolism were altered in non-responders, suggesting that these pathways are affected more in cases of poor response to treatment. In contrast, more metabolism-related proteins were affected in responder syndicating that a metabolic change might be required for or associated with a favorable response.

## Conclusions

This is one of only a few mass spectrometry-based proteomics studies, which has attempted to identify a plasma protein fingerprint for prediction of antidepressant treatment response. A recent mass spectrometry profiling study identified a 5-component biomarker panel including apolipoprotein B that was capable of distinguishing MDD and control groups with good accuracy (Lee et al., 2016). A multiplex immunoassay profiling study confirmed that this protein may be involved in the pathophysiology of depression (Frye et al., 2015). Another multiplex immunoassay study found a 9-component panel predictive of response to different antidepressants and one of these proteins (apolipoprotein A-IV) was also identified in this study (Chan et al., 2016).

In our study, LC-MS/MS profiling resulted in identification of 29 proteins at baseline that were present at different levels in those patients who had a subsequent favorable response to treatment. Most of these proteins were associated with either metabolism or immune pathways and 9 of these proteins showed significant changes during the 6-week treatment period. However, we have to note that treatment was not systematically varied but selected according to the psychiatrist (and patient's) choices with the aim to continuously optimize antidepressant treatment. There was also no washout period before treatment was started. Thus, the results should be considered as preliminary and warrant further investigation and validation. Nevertheless, it should be stressed that the small sample size analyzed in this study renders the results as preliminary, thus requiring follow up validation work using larger cohorts on repeated occasions. This will enhance generalization of the findings. Furthermore, the different treatment types may influence the response rate and this was not accounted for in this study. Nevertheless, these results represent a promising first step toward the development of a clinical test for biologically informed treatment decisions of psychiatrists and clinicians to improve treatment outcomes and management of patients suffering from MDD. In turn, this should lead to improved patient outcomes and add novel biological targets.

## Author contributions

CT and DM conceived and designed the study. MI, SK, SL, and FH organized sample collection and provided clinical support. DM carried out proteomic experiments, analyzed and interpreted the data. PG helped on data analyses and wrote the first draft of the manuscript. GM helped on proteomic data analyses helped to carry out the experiments. All authors contributed to data discussion, read and approved the final version of this manuscript.

### Conflict of interest statement

The authors declare that the research was conducted in the absence of any commercial or financial relationships that could be construed as a potential conflict of interest.

## Footnotes

^1^www.who.int/mediacentre/factsheets/fs369/en/

## References

[B1] Alsaif M. Guest P. C. Schwarz E. Reif A. Kittel-Schneider S. Spain M.. (2012). Analysis of serum and plasma identifies differences in molecular coverage, measurement variability, and candidate biomarker selection. Proteomics Clin. Appl. 6, 297–303. 10.1002/prca.201100061 22641612

[B2] Bernstein H. G. Stricker R. Dobrowolny H. Steiner J. Bogerts B. Trübner K.. (2013). Nardilysin in human brain diseases: both friend and foe. Amino Acids 45, 269–278. 10.1007/s00726-013-1499-8 23604405

[B3] Bromet E. Andrade L. H. Hwang I. Sampson N. A. Alonso J. de Girolamo G.. (2011). Cross-national epidemiology of DSM-IV major depressive episode. BMC Med. 9:90. 10.1186/1741-7015-9-90 21791035 PMC3163615

[B4] Chan M. K. Cooper J. D. Bot M. Birkenhager T. K. Bergink V. Drexhage H. A.. (2016). Blood-based immune-endocrine biomarkers of treatment response in depression. J. Psychiatr. Res. 83, 249–259. 10.1016/j.jpsychires.2016.08.020 27693950

[B5] Chan M. K. Gottschalk M. G. Haenisch F. Tomasik J. Ruland T. Rahmoune H.. (2014). Applications of blood-based protein biomarker strategies in the study of psychiatric disorders. Prog. Neurobiol. 122, 45–72. 10.1016/j.pneurobio.2014.08.002 25173695

[B6] Chan M. K. Krebs M. O. Cox D. Guest P. C. Yolken R. H. Rahmoune H.. (2015). Development of a blood-based molecular biomarker test for identification of schizophrenia before disease onset. Transl. Psychiatry 5:e601. 10.1038/tp.2015.91 26171982 PMC5068725

[B7] Frye M. A. Nassan M. Jenkins G. D. Kung S. Veldic M. Palmer B. A.. (2015). Feasibility of investigating differential proteomic expression in depression: implications for biomarker development in mood disorders. Transl. Psychiatry 5:e689. 10.1038/tp.2015.185 26645624 PMC5068585

[B8] Giménez-Palop O. Coronas R. Cobo J. Gallart L. Barbero J. D. Parra I.. (2012). Fasting plasma peptide YY concentrations are increased in patients with major depression who associate weight loss. J. Endocrinol. Invest. 35, 645–648. 10.3275/8180 22183081

[B9] Guest P. C. Guest F. L. Martins-de Souza D. (2015). Making sense of blood-based proteomics and metabolomics in psychiatric research. Int. J. Neuropsychopharmacol. 19:pyv138 10.1093/ijnp/pyv138 PMC4926797 26721951

[B10] Haenisch F. Alsaif M. Guest P. C. Rahmoune H. Yolken R. H. Dickerson F.. (2015). Multiplex immunoassay analysis of plasma shows differences in biomarkers related to manic or mixed mood states in bipolar disorder patients. J. Affect. Disord. 185, 12–16. 10.1016/j.jad.2015.05.065 26142689

[B11] Hamilton M. (1960). A rating scale for depression. J. Neurol. Neurosurg. Psychiatr. 23, 56–62. 10.1136/jnnp.23.1.56 14399272 PMC495331

[B12] Hennings J. M. Owashi T. Binder E. B. Horstmann S. Menke A. Kloiber S.. (2009). Clinical characteristics and treatment outcome in a representative sample of depressed inpatients-findings from the Munich Antidepressant Response Signature (MARS) project. J. Psychiatr. Res. 43, 215–229. 10.1016/j.jpsychires.2008.05.002 18586274

[B13] Holzer P. Reichmann F. Farzi A. (2012). Neuropeptide Y peptide YY and pancreatic polypeptide in the gut-brain axis. Neuropeptides 46, 261–274. 10.1016/j.npep.2012.08.005 22979996 PMC3516703

[B14] Jordan W. Dobrowolny H. Bahn S. Bernstein H. G. Brigadski T. Frodl T.. (2016). Oxidative stress in drug-naïve first episode patients with schizophrenia and major depression: effects of disease acuity and potential confounders. Eur. Arch. Psychiatry Clin. Neurosci. 10.1007/s00406-016-0749-7. [Epub ahead of print]. 27913877

[B15] Kim E. Y. Lee M. Y. Kim S. H. Ha K. Kim K. P. Ahn Y. M. (2017). Diagnosis of major depressive disorder by combining multimodal information from heart rate dynamics and serum proteomics using machine-learning algorithm. Prog. Neuropsychopharmacol. Biol. Psychiatry 76, 65–71. 10.1016/j.pnpbp.2017.02.014 28223106

[B16] Lee M. Y. Kim E. Y. Kim S. H. Cho K. C. Ha K. Kim K. P.. (2016). Discovery of serum protein biomarkers in drug-free patients with major depressive disorder. Prog. Neuropsychopharmacol. Biol. Psychiatry 69, 60–68. 10.1016/j.pnpbp.2016.04.009 27105922

[B17] Liu X. Zheng P. Zhao X. Zhang Y. Hu C. Li J.. (2015). Discovery and validation of plasma biomarkers for major depressive disorder classification based on liquid chromatography-mass spectrometry. J. Proteome Res. 14, 2322–2330. 10.1021/acs.jproteome.5b00144 25784130

[B18] Lopez-Vilchez I. Serra-Millas M. Navarro V. Rosa Hernandez M. Villalta J. Diaz-Ricart M. (2014). Prothrombotic platelet phenotype in major depression: down regulation by antidepressant treatment. J. Affect. Disord. 159, 39–45. 10.1016/j.jad.2014.02.022 24679387

[B19] Maccarrone G. Rewerts C. Lebar M. Turck C. W. Martins-de-Souza D. (2013). Proteome profiling of peripheral mononuclear cells from human blood. Proteomics 13, 893–897. 10.1002/pmic.201200377 23281267

[B20] Martins-de-Souza D. Maccarrone G. Ising M. Kloiber S. Lucae S. Holsboer F.. (2014). Plasma fibrinogen: now also an antidepressant response marker? Transl. Psychiatry 4:e352. 10.1038/tp.2013.129 24473443 PMC3905236

[B21] Martins-de-Souza D. Solari F. A. Guest P. C. Zahedi R. P. Steiner J. (2015). Biological pathways modulated by antipsychotics in the blood plasma of schizophrenia patients and their association to a clinical response. NPJ Schizophr. 1:15050. 10.1038/npjschz.2015.50 27336048 PMC4849468

[B22] Śliwińska-Mossoń M. Borowiecka K. Milnerowicz H. (2013). Neuropeptides, Y, YY, PP and their clinical significance. Postepy Hig. Med. Dosw. (Online) 67, 631–636. 10.5604/17322693.1058890 24018426

[B23] Pompili M. Venturini P. Palermo M. Stefani H. Seretti M. E. Lamis D. A. (2013). Mood disorders medications: predictors of nonadherence-review of the current literature. Expert Rev. Neurother. 13, 809–825. 10.1586/14737175.2013.811976 23898852

[B24] Ruland T. Chan M. K. Stocki P. Grosse L. Rothermundt M. Cooper J. D.. (2016). Molecular serum signature of treatment resistant depression. Psychopharmacology (Berl). 233, 3051–3059. 10.1007/s00213-016-4348-0 27325393

[B25] Saia-Cereda V. M. Cassoli J. S. Schmitt A. Falkai P. Nascimento J. M. Martins-de-Souza D. (2015). Proteomics of the corpus callosum unravel pivotal players in the dysfunction of cell signaling, structure, and myelination in schizophrenia brains. Eur. Arch. Psychiatry Clin. Neurosci. 265, 601–612. 10.1007/s00406-015-0621-1 26232077

[B26] Schwarz E. Guest P. C. Rahmoune H. Harris L. W. Wang L. Leweke F. M.. (2012a). Identification of a biological signature for schizophrenia in serum. Mol. Psychiatry 17, 494–502. 10.1038/mp.2011.42 21483431

[B27] Schwarz E. Guest P. C. Steiner J. Bogerts B. Bahn S. (2012b). Identification of blood-based molecular signatures for prediction of response and relapse in schizophrenia patients. Transl. Psychiatry 2:e82 10.1038/tp.2012.3 22832819 PMC3309553

[B28] Schwarz E. Steiner J. Guest P. C. Bogerts B. Bahn S. (2015). Investigation of molecular serum profiles associated with predisposition to antipsychotic-induced weight gain. World J. Biol. Psychiatry 16, 22–30. 10.3109/15622975.2013.817685 24001020

[B29] Stelzhammer V. Haenisch F. Chan M. K. Cooper J. D. Steiner J. Steeb H. (2014). Proteomic changes in serum of first onset, antidepressant drug-naïve major depression patients. Int. J. Neuropsychopharmacol. 17, 1599–1608. 10.1017/S1461145714000819 24901538

[B30] Tomasik J. Schwarz E. Lago S. G. Rothermundt M. Leweke F. M. van Beveren N. J.. (2016). Pretreatment levels of the fatty acid handling proteins H-FABP and CD36 predict response to olanzapine in recent-onset schizophrenia patients. Brain Behav. Immun. 52, 178–186. 10.1016/j.bbi.2015.10.019 26541453

[B31] van Beveren N. J. Schwarz E. Noll R. Guest P. C. Meijer C. de Haan L.. (2014). Evidence for disturbed insulin and growth hormone signaling as potential risk factors in the development of schizophrenia. Transl. Psychiatry 4:e430. 10.1038/tp.2014.52 25158005 PMC4150237

[B32] Yang Y. Chen J. Liu C. Fang L. Liu Z. Guo J.. (2016). The extrinsic coagulation pathway: a biomarker for suicidal behavior in major depressive disorder. Sci. Rep. 6:32882. 10.1038/srep32882 27605454 PMC5015115

[B33] Young J. J. Bruno D. Pomara N. (2014). A review of the relationship between proinflammatory cytokines and major depressive disorder. J. Affect. Disord. 169, 15–20. 10.1016/j.jad.2014.07.032 25128861

